# Intraoperative Stereotactic Arteriography in Complex Cervical Spine Surgery

**DOI:** 10.7759/cureus.56783

**Published:** 2024-03-23

**Authors:** Luke Mugge, Danielle D Dang, Mateo Ziu, Andrew Fanous

**Affiliations:** 1 Department of Neurosurgery, Inova Neuroscience and Spine Institute, Falls Church, USA; 2 Department of Neurosurgery, Inova Neuroscience and Spine Institute, Fairfax, USA

**Keywords:** vertebral artery, cervical spine, angiogram, stereotaxis, o-arm

## Abstract

Intra-operative navigation has revolutionized spinal instrumentation. The O-arm (a mobile X-ray system; Medtronic, Minneapolis, MN) is uniquely capable of enabling visualization of the spine in axial planes. The application of this technology is wide yet underutilized in terms of its capacity to image spinal vascular anatomy. We completed a retrospective chart review of the following case studies. A 24-year-old neurologically intact female presented with a Jefferson fracture without vertebral artery dissection after a motor vehicle accident. After the failure of conservative management due to pseudoarthrosis, the patient opted for fusion. Prior to the procedure, bilateral 5 French femoral sheaths were placed. After exposure, intraarterial (IA) contrast was injected prior to the O-arm spin to visualize both vertebral arteries, which were stretched and adjacent to a mobile boney segment. In the second case, a 71-year-old male presented with right shoulder pain and a flaccid left deltoid secondary to a large enhancing epidural lesion spanning C4-C7. Further work-up confirmed a diagnosis of metastatic intrahepatic cholangiocarcinoma. Prior to resection with cervical spinal stabilization, a right radial artery 4 French Glidesheath was placed. Prior to the O-arm spin, the right vertebral artery was selected, and intravenous contrast was injected to permit visualization of the vertebral artery, which was encased within the tumor and at significant risk for iatrogenic injury. Both patients tolerated the endovascular and spinal procedures well without vertebral artery injury. This is the first series to report the effective use of the O-arm for improved visualization of vascular anatomy during surgery for cervical spinal trauma and oncology.

## Introduction

Vertebral artery injury (VAI) is a constant concern with the placement of cervical spinal instrumentation. While the rate of VAI may be as low as 0.07% in some studies, it is as high as 2% in cases of atlanto-axial fixation [1,2]. The rates of ensuing neurological injury or even death from fatal brainstem infarcts can be as high as 10% [1]. Essential to the prevention of such complications is having a robust understanding of the patient’s individual vascular anatomy, recognition of deviations from anticipated norms, and anticipation of necessary compensatory surgical maneuvers [3]. Causes of VAI predominately include retraction, drilling, and instrumentation [4]. In many cases, salvage is not possible, and endovascular sacrifice of the injured vertebral artery (VA) is required [5]. As such, primary efforts should be aimed at prevention, rather than repair, of VAI. In this study, we present a technical description of utilizing the O-arm (a mobile X-ray system; Medtronic, Minneapolis, MN) in combination with intra-arterial (IA) contrast administration for the visualization of the VA during cervical instrumentation. This technique was employed in the context of both cervical spine trauma and tumor resection. In both cases, our technique was effective in intraoperative identification of the VA, in permitting safe instrumentation and in avoiding VAI.

## Case presentation

Case 1: trauma

Presentation

**Figure 1:**
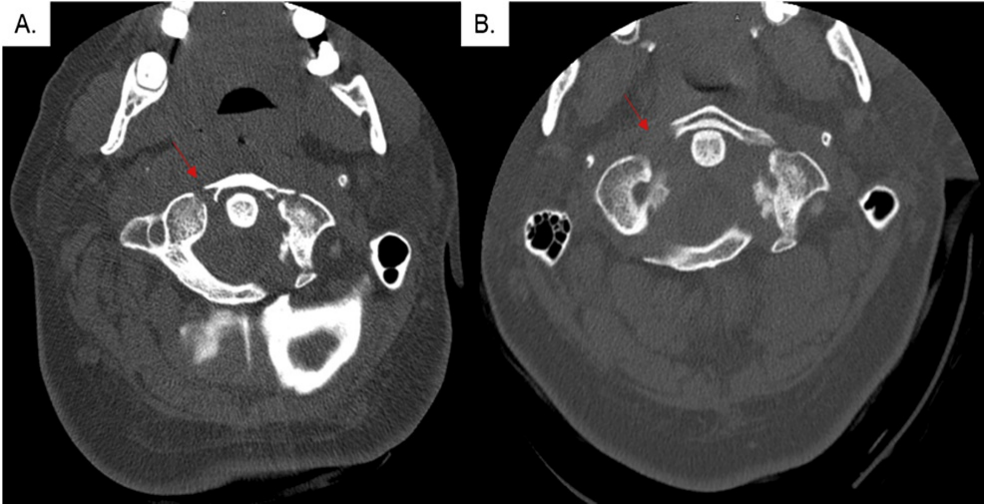
Pre-operative CT of the cervical fracture A) Initial axial non-contrast cervical spine CT centered on the C1 vertebra demonstrates Jefferson fractures involving the anterior (red arrow) and posterior rings, with minimal displacement; B) Follow-up axial non-contrasted cervical spine CT demonstrates worsening displacement and widening of the fracture lines (red arrow).

A 24-year-old woman presented with neck pain after a motor vehicle accident. She was neurologically intact on examination. Non-contrasted computed tomography (CT) of the cervical spine demonstrated a Jefferson fracture. After one month of conservative management with collar-immobilization, the fracture was noted to have increased displacement, from 1.5 mm to 3.8 mm (Figure 1).

**Figure 2:**
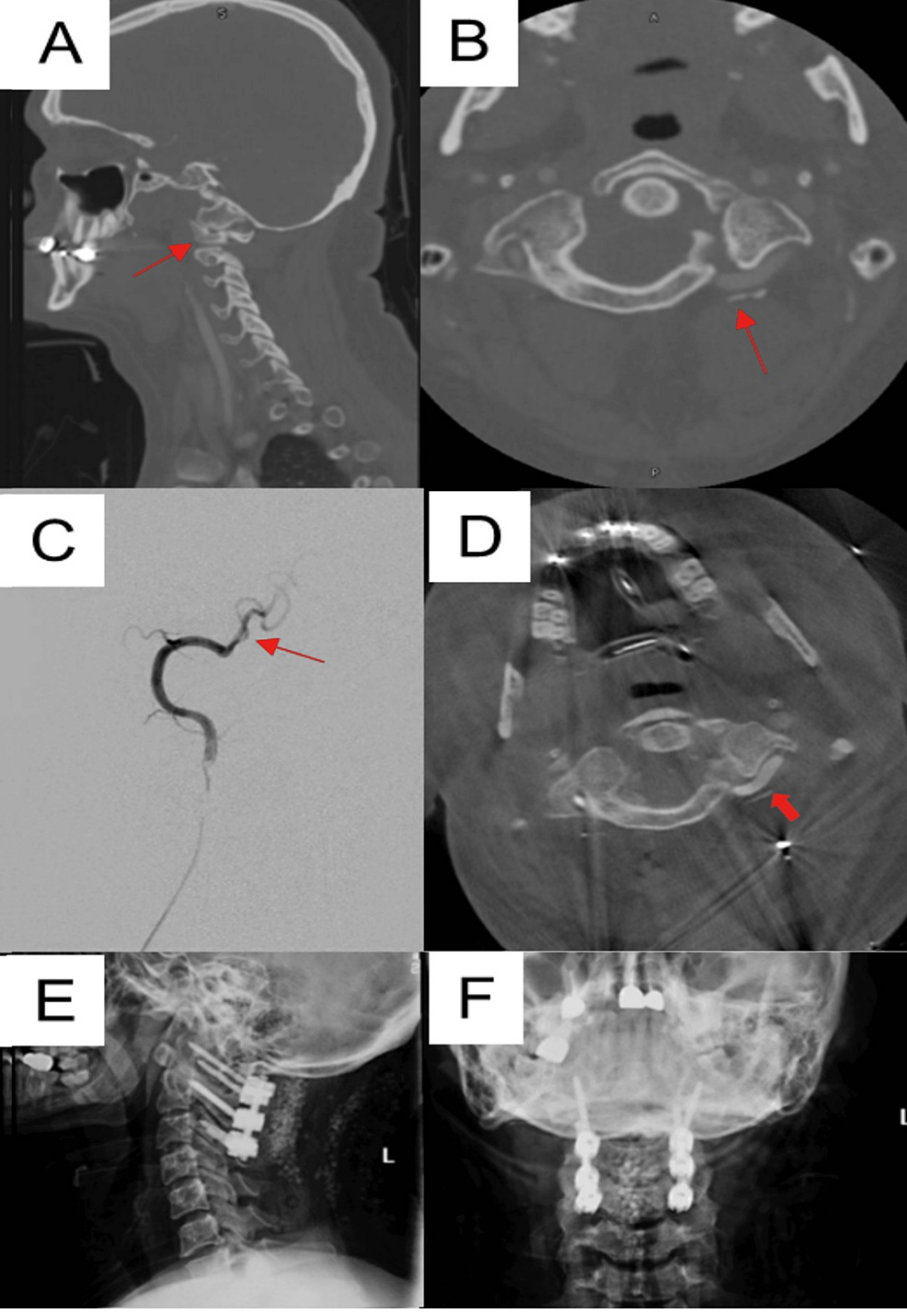
Trauma case A) Pre-operative sagittal CTA; B) pre-operative axial CTA; C) Intra-operative digital subtraction angiogram demonstrating catheter placement within the proximal right vertebral artery; D) Intra-operative computed tomography angiography (CTA) using O-arm (Medtronic, Minneapolis, MN) demonstrating the visualization of distorted vertebral arteries (red arrow); E) Post-operative lateral X-ray; F) Post-operative anteroposterior (A/P) X-ray.

After discussing the various options with the patient, she opted for surgical stabilization. During the pre-operative period, a CT angiogram (CTA) was obtained, which demonstrated intact but significantly displaced and stretched VA over the sulcus arteriosus bilaterally (Figures 2A-2B).

Surgical Procedure

The patient was initially taken to the interventional radiology suite, where bilateral 5-French femoral artery sheaths were placed, followed by the placement of bilateral 5-French H1H catheters into the proximal vertebral artery (VA) (Figure 2C). The patient was subsequently taken to the operating room, where endotracheal intubation was undertaken and the patient was placed in a prone position. Following surgical exposure, the O-arm navigation tracker was attached to the C2 spinous process. The posterior C1 arch was noted to be abnormally mobile. Intra-arterial contrast was then injected through the bilateral selective VA catheters, followed by immediate O-arm stereotactic navigation spin (Figure 2D). This enabled clear visualization and localization of the bilateral displaced VA during the operation. Placement of C1, C2, and C3 screws was subsequently successfully performed under stereotactic guidance while avoiding the bilateral distorted VA, which were easily visualized during the instrumentation process. The remainder of the surgery, including rod placement and arthrodesis, proceeded uneventfully, and the patient tolerated the procedure well. She remained neurologically intact post-operatively. She was discharged home on the second post-operative day. Post-operative imaging demonstrated excellent placement of the instrumentation (Figures 2E-2F).

Case 2: tumor

Presentation

**Figure 3:**
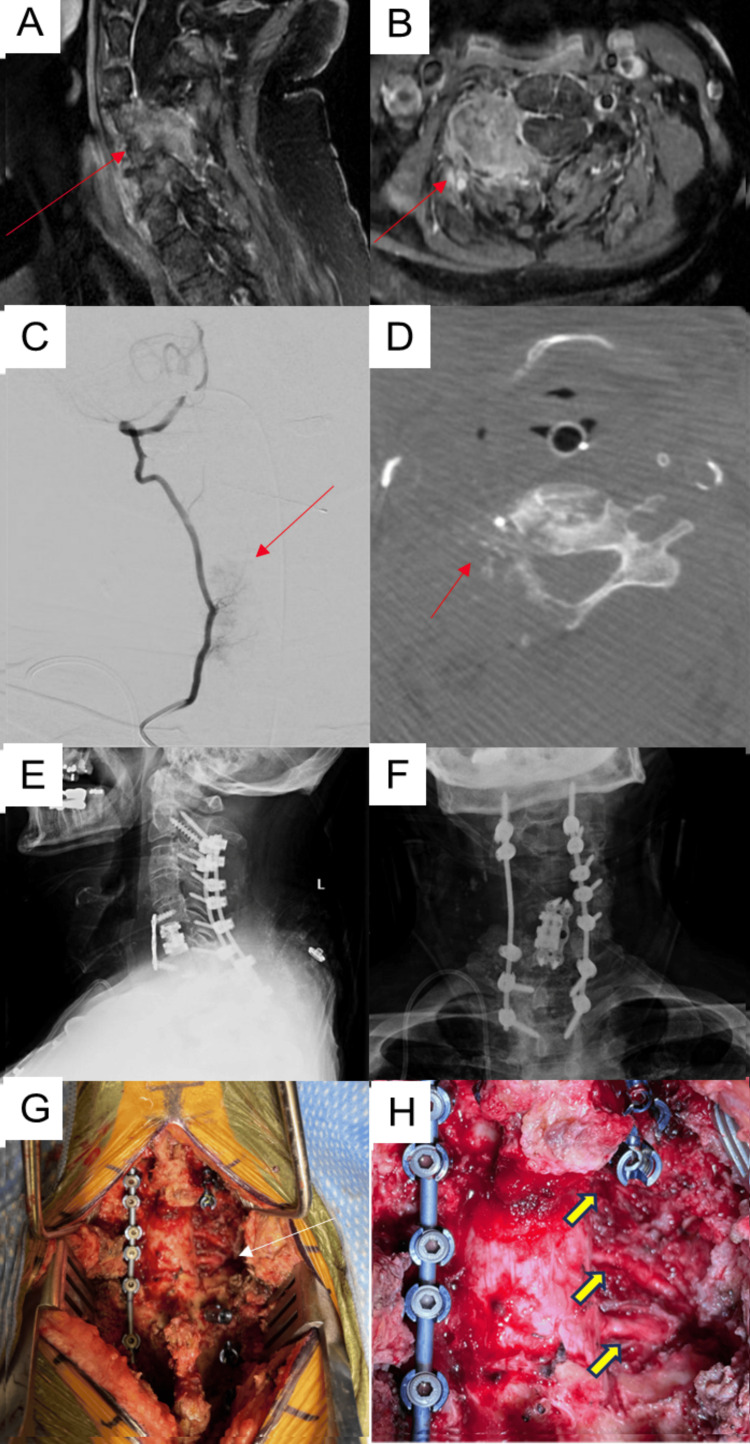
Tumor case A) Pre-operative sagittal T1-weighted post-contrast MRI of the cervical spine; B) Pre-operative axial T1-weighted post-contrast MRI of the cervical spine; C) Intra-operative sagittal angiogram demonstrating catheter placement within the proximal right vertebral artery; D) Intra-operative CTA using O-arm (Medtronic, Minneapolis, MN) demonstrating the complete envelopment of the right vertebral artery by tumor (red arrow); E) Post-operative lateral X-ray; F) Post-operative A/P X-ray; G) Intra-operative view at low magnification following tumor resection; H) Intra-operative view higher magnification following tumor resection demonstrating complete tumor resection and skeletonized thecal sac and nerve roots on the right side (yellow arrows).

A 71-year-old man presented with several months of worsening right neck pain with radiation to the right arm, which remained refractory to conservative treatment. On examination, he had profound proximal right arm weakness, as well as significant signs of myelopathy and hyperreflexia. Magnetic resonance imaging (MRI) of the cervical spine with and without contrast administration revealed a large heterogeneously-enhancing tumor involving the anterior and posterior cervical spine from C3-C6, with compression of the spinal cord and the right C4, C5, and C6 nerve roots (Figures 3A-3B). Further workup revealed evidence of moderately differentiated metastatic intrahepatic cholangiocarcinoma. Following a discussion of the various options with the patient and his family, they opted for surgical intervention to decompress the spinal cord and the cervical nerve roots. A two-staged procedure was planned consisting of a C6 corpectomy and C5-C7 anterior arthrodesis, followed by C3-C6 laminectomy and right facetectomy with C2-T2 posterior instrumented fusion. During the pre-operative planning period, CTA of the cervical spine was obtained, which demonstrated patency of the right VA in spite of its engulfment by the tumor.

Surgical Procedure

After completion of the first stage of the surgery, a right radial artery 4-French Glidesheath Slender was placed under ultrasound guidance. The following day, the patient was taken to the operating room for the second stage of the operation. The patient was intubated and placed in a prone position. Following surgical exposure, the O-arm navigation tracker was attached to the C2 spinous process. The right VA was selected, and a trans-radial right vertebral arteriogram was performed with an intra-arterial contrast injection. This was followed by immediate O-arm stereotactic navigation spin, which permitted clear intraoperative visualization and localization of the right VA (Figures 3C-3D). Resection of the tumor, which was completely engulfing and distorting the right vertebral artery, proceeded under microscopic and stereotactic visualization. The spinal cord and the right C4, C5, and C6 nerve roots were decompressed (Figures 3G-3H). The ability to accurately localize the right VA during tumor resection allowed the safe removal of the tumor from around the VA without causing any arterial injury. Successful instrumentation then proceeded from C2 through T2 under stereotactic guidance, followed by rod placement and arthrodesis. The patient tolerated the procedure well. He experienced an improvement in his right proximal arm weakness post-operatively. He was discharged to a rehabilitation facility on the sixth post-operative day. Post-operative imaging demonstrated excellent placement of the instrumentation (Figures 3E-3F).

## Discussion

In our current case series, we report the first-ever use of O-arm stereotactic navigation with intra-arterial contrast administration for the treatment of a traumatic spinal fracture, as well as the first-ever reported use of such technology for the surgical treatment of a cervical spine neoplasm. This technology permits the accurate visualization and localization of the vertebral arteries, whose course is distorted by these pathologies. In the case of our patient with the Jefferson fracture, the bilateral VAs were stretched by the displaced bony fragments, thus complicating the placement of C1 lateral mass screws and placing the VA at high risk of iatrogenic injury during instrumentation. In the case of our patient with the cervical neoplasm, the right VA was completely encased by the tumor, and our technique permitted successful tumor resection from around the artery without the need to sacrifice it and without placing it at risk of injury during surgery. Our study has demonstrated the practicality, utility, effectiveness, and safety of intraoperative stereotactic arteriography in the context of complex spine anatomy.

The introduction of the O-arm for stereotactic neuronavigation has revolutionized the field of neurological and spinal surgery [6-9]. In addition to its originally intended purpose of providing stereotactic navigation for instrumentation placement in the spine, the O-arm has been recently used for the assessment of vascular anatomy. For instance, in 2019, Torne et al. demonstrated the effectiveness of the O-arm in visualizing intra-cranial aneurysms [10]. In 2020, Pavlov et al. proposed the idea of using the O-arm to visualize the VA in spine surgery [11]. In that theoretical study, Pavlov et al. hypothesized that the unique technology of the O-arm could permit simultaneous visualization of the bony and vascular anatomy in the cervical spine, assuming IA contrast was administered, thereby theoretically lowering the risk of vertebral artery injury down to zero. In 2021, Kiessling et al. described the utilization of intraoperative O-arm-based vascular imaging wherein intra-venous contrast was administered after which the scan was timed to optimally visualize the arterial system [12]. This case examined a 54-year-old man with an unstable C2 fracture and a right vertebral artery occlusion [12]. That technology significantly differs from ours in that it employs venography rather than IA contrast administration, making our case series the first ever to report selective arteriography in combination with the O-arm for the surgical treatment of spine patients.

A number of technical nuances are worth mentioning with regard to the use of intraoperative stereotactic arteriography in spine surgery. Firstly, since patients undergoing spine surgery tend to be placed in a prone surgical position, placement of the intra-arterial sheath and catheter needs to occur prior to patient positioning, because placement of such devices in the prone position is simply not feasible. While this can occur in any setting, we recommend placement of the intra-arterial sheath in the interventional radiology/endovascular suite. In hospitals with hybrid capabilities, placement of the intra-arterial devices may also take place in the hybrid operating room after anesthesia induction and prior to patient positioning. However, this is likely to contribute to significant operative delays and to increased anesthesia time for the patient. Secondly, our case series has demonstrated that both femoral and radial access are equally effective in selective intra-operative VA arteriography. Having both options can be useful in cases where the anatomy of the aortic arch is partial to one approach over the other. Thirdly, it must be stressed that successful intraoperative stereotactic arteriography requires the administration of intra-arterial contrast immediately prior to the O-arm spin. As such, placement of the O-arm navigation tracker, positioning of the O-arm machine and proper localization of the correct spinal segments must all take place prior to IA contrast administration. Any delay between injection of the contrast medium and spinning of the O-arm is likely to result in washout of the contrast and inability to properly visualize the arteries.

There are a number of risks associated with our technique. Catheter displacement or migration may occur prior to entering the operating room and can result in vascular complications. Another risk is thrombosis or arterial occlusion secondary to the presence of endovascular devices within the relatively small VA. Meticulous securing of the arterial sheath and catheter, as well as heparinization of the entire system, are paramount in avoiding such potential complications. Our current study is subject to all the limitations intrinsic to the case series. We report only two cases with different pathologies. Therefore, larger studies are needed to confirm the utility and safety of our technique.

## Conclusions

Aberrant or distorted vertebral artery anatomy, which results from a myriad of pathologies within the cervical spine including trauma or tumors, can result in severe and potentially fatal iatrogenic injury to these vessels during spine surgery. Intraoperative stereotactic arteriography in the context of such complex cases is safe, practical, and effective in avoiding such complications. This technique permits the visualization of the vertebral arteries during surgery, which in turn allows for safe instrumentation and the ability to remove tumors encasing these arteries while avoiding any iatrogenic injury to these critical vessels.
